# Measuring Locomotor Activity and Behavioral Aspects of Rodents Living in the Home-Cage

**DOI:** 10.3389/fnbeh.2022.877323

**Published:** 2022-04-07

**Authors:** Christian J. M. I. Klein, Thomas Budiman, Judith R. Homberg, Dilip Verma, Jaap Keijer, Evert M. van Schothorst

**Affiliations:** ^1^Human and Animal Physiology, Wageningen University and Research, Wageningen, Netherlands; ^2^TSE Systems GmbH, Berlin, Germany; ^3^Department of Cognitive Neuroscience, Donders Institute for Brain, Cognition and Behavior, Radboud University Medical Center, Nijmegen, Netherlands

**Keywords:** locomotor activity, behavior, home-cage, rodents, 3Rs, phenotyping, animal tracking

## Abstract

Automatization and technological advances have led to a larger number of methods and systems to monitor and measure locomotor activity and more specific behavior of a wide variety of animal species in various environmental conditions in laboratory settings. In rodents, the majority of these systems require the animals to be temporarily taken away from their home-cage into separate observation cage environments which requires manual handling and consequently evokes distress for the animal and may alter behavioral responses. An automated high-throughput approach can overcome this problem. Therefore, this review describes existing automated methods and technologies which enable the measurement of locomotor activity and behavioral aspects of rodents in their most meaningful and stress-free laboratory environment: the home-cage. In line with the Directive 2010/63/EU and the 3R principles (replacement, reduction, refinement), this review furthermore assesses their suitability and potential for group-housed conditions as a refinement strategy, highlighting their current technological and practical limitations. It covers electrical capacitance technology and radio-frequency identification (RFID), which focus mainly on voluntary locomotor activity in both single and multiple rodents, respectively. Infrared beams and force plates expand the detection beyond locomotor activity toward basic behavioral traits but discover their full potential in individually housed rodents only. Despite the great premises of these approaches in terms of behavioral pattern recognition, more sophisticated methods, such as (RFID-assisted) video tracking technology need to be applied to enable the automated analysis of advanced behavioral aspects of individual animals in social housing conditions.

## Introduction

Historically, animals’ behavior was monitored, assessed and quantified manually by an experienced human observer in real-time (Altmann, 1974). This process is very time- and labor-intensive, prevents large-scale and high-throughput studies, is mostly restricted to daytime scoring, subjective to the human observer and thus prone to (human) bias (Levitis et al., 2009). This required the development of alternative, automated methods to make (behavioral) phenotyping more rapid, objective, and consistent within and across laboratories, aiming to increase reproducibility and replicability of research outcomes (Kafkafi et al., 2018). Automatization will also help to standardize experiments, which are impacted by heterogeneity between laboratories (Crabbe et al., 1999; Crawley, 1999; Muller et al., 2016) and personnel (Kennard et al., 2021). Automatization is especially relevant during social animal experimentations, which stimulate very complex and rich behavioral profiles challenging to the human eye. One well-established way to increase standardization and reduce (bias from) animal handling is to study animals in their “living room”: the home-cage. So far, the introduction of automatization as well as of computational ethology has led to an enormous number of different methods to study behavioral and physiological traits in various animals and experimental set-ups (Anderson and Perona, 2014; Dell et al., 2014; Voikar and Gaburro, 2020). This review focusses on rodents and aims to give an overview of current technologies and methods which enable researchers to automatically study rodents’ locomotor activity and behavioral traits, highlighting their individual strengths and limitations. It includes electrical capacitance, radio-frequency identification (RFID), infrared (IR) beams, force plates, and (RFID-assisted) video tracking technology. Since the Directive 2010/63/EU recommends the housing of social animals in social conditions during experimentation for animal welfare reasons, this review furthermore evaluates the suitability and limitations of the described technologies to study socially housed rodents either in their home-cage or in a social arena. For the purpose of this review, any cage environment in which multiple (at least 2) rodents can be housed under minimally stressed conditions for a long duration (several weeks to months) with appropriate bedding and nesting material as well as access to feed and drink is taken as the home cage or social arena. At the end, this review will also provide insights into current developments in the field of multiple animal tracking as well as possible future directions in the field.

## Measuring Voluntary Locomotor Activity

### Electrical Capacitance

**FIGURE 1:**
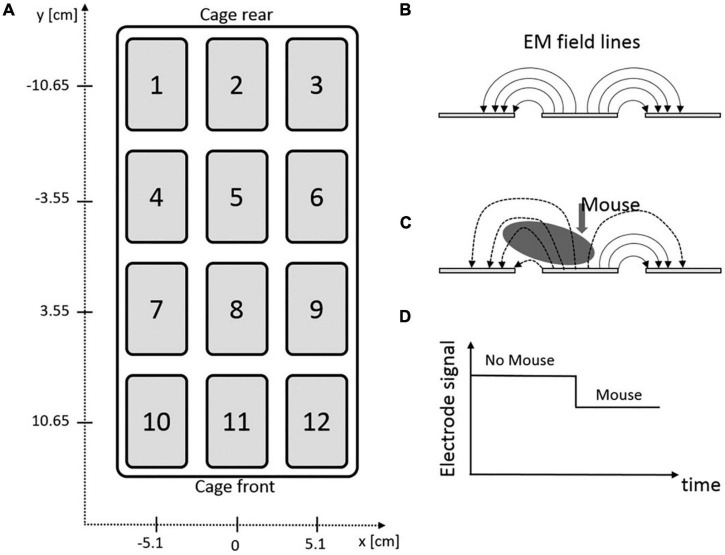
DVC sensor plate of Tecniplast. **(A)** Graphical illustration of electrical board containing 12 electrodes. **(B)** Side-view of three adjacent electrodes and the corresponding electromagnetic (EM) field lines. **(C)** Effect of animal’s presence on the EM field lines. **(D)** Impact of animal’s presence on electrode signal output (Iannello, 2019).

Measuring an animal’s activity can be done by electrical capacitance technology. This technology comprises several electrodes embedded in an electronic sensing board (Figure 1), which is installed underneath the home-cage. The animal’s presence changes the electromagnetic field emitted by these electrodes. Thereby, the exact position (with spatial resolution of 1 mm) and trajectory can be identified based on capacity variation [with temporal resolution of 4 hertz (Hz)]. The sensing board sends its raw data to an associated software and computer infrastructure, which enables the researcher to additionally analyze distance traveled, average speed, position distribution, and activity density of the animal. The activity metrics show comparable results when benchmarked against video-recording technology (Iannello, 2019). This board was developed as part of the *Digital Ventilated Cage* (DVC) monitoring system (Tecniplast, Buguggiate, Italy), allowing fully automated, 24/7, non-invasive, real-time activity monitoring and traceability of individually housed mice. It requires only modest computational power resulting in a small data footprint per unit. It is highly scalable, allowing arbitrary numbers of home-cages to be monitored simultaneously. DVC-derived datasets can be used subsequently for a deeper analysis of several activity metrics in individual-housed mice (Shenk et al., 2020). However, this system does not support the analysis of ethologically relevant behavioral patterns (grooming, rearing, climbing etc.) which makes it less suitable for phenotyping and behavioral studies. It is currently also designed for the use of mice only. Whereas multiple animals can be housed in one home-cage to monitor group activity (Pernold et al., 2019), the full potential of the technology relies on individually housed conditions. This makes this system currently unable to study social interaction and behavior. Since it was originally developed as a component of the DVC system, it cannot be integrated in automated monitoring systems of other vendors. In conclusion, the sensor plate is a useful module within the DVC system aiming to improve animals’ health monitoring and facility management. It allows monitoring of overall activity, but the limited behavioral pattern recognition makes this system less suitable for more sophisticated phenotyping and behavioral studies, especially in group-housed settings.

### Radio-Frequency Identification

**FIGURE 2:**
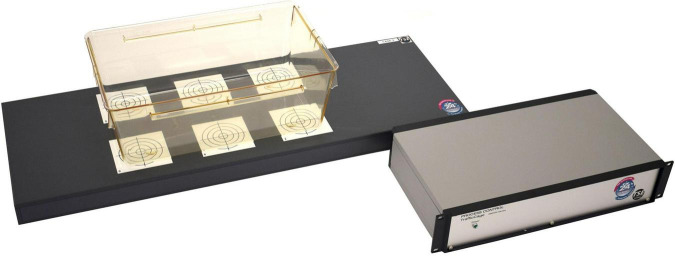
The TraffiCage (TSE Systems) uses a RFID matrix underneath the home-cage to track multiple animals in the home-cage.

Locomotion activity can also be measured using radio-frequency identification (RFID) technology. RFID uses radio-waves to wirelessly identify and track specific tags, which can be attached to or inserted into objects and animals. The technology consists of four elements: tags, readers, antennas, and a computer network for data handling. Passive RFID tags do not require an internal power supply (battery)—in contrast to active tags—since they are powered *via* the radio waves emitted by the antennas. This reduces the overall size of the RFID tag, which makes passive tags more suitable for implantation in small laboratory animals. The RFID tag is activated once it is in the range of an RFID antenna and thereby sends its unique ID code to the RFID reader. Depending on the type of tag, additional information (strain, age, etc.) can be stored and conveyed. It allows also for physiological characteristics (i.e., peripheral body temperature) to be measured additionally (Unified Information Devices Inc., Lake Villa, United States) (Winn et al., 2021). When implanted into the animals, individual animals are tracked and identified within the home-cage or any other experimental unit. The benefits of RFID technology have inspired researchers to develop the IntelliCage (TSE Systems, Berlin, Germany) which enables the study of complex behaviors in socially interacting mice and rats living in a stress-free cage environment without human interference (Lipp et al., 2005; Kiryk et al., 2020). The IntelliCage consists of four operant conditioning corners and allows for several, longitudinal (social) behavioral and cognitive test batteries in a meaningful and social living environment. Furthermore, it can serve as a core component of a new automated multi-dimensional phenotyping paradigm: the PhenoWorld (TSE Systems). The PhenoWorld supports behavioral, cognitive, metabolic, and physiological measurements in an ethological meaningful multi-component living environment stimulating rodents to display their species-specific natural social behavior (Castelhano-Carlos et al., 2014, 2017). Others applied a similar approach to study groups of rodents in a semi-naturalistic environment by installing RFID antennas at strategically relevant locations within multiple living quarters (Lewejohann et al., 2009; Howerton et al., 2012; Puścian et al., 2016; Linnenbrink and von Merten, 2017; Habedank et al., 2021). However, since RFID antennas are usually not distributed equally within those (multi-) cage environments, but rather at strategically interesting locations, only cross trajectories and cross activity of individual animals can be measured. To circumvent this and to get a more accurate picture of the activity and trajectories of individual rodents, a non-commercial passive RFID system based on ultra-high frequency was developed suitable for standard home-cage applications in rodents (Catarinucci et al., 2014). *In vivo* validation against IR-beams (see “Infrared Beams”) and a well-established video-tracking system showed a strong correlation regarding positional data and total activity (Catarinucci et al., 2014; Macrì et al., 2015). This design has been adopted by the industry resulting in commercially available systems consisting of a RFID antenna matrix underneath the home-cage, in which each antenna emits a confined electromagnetic field (Figure 2). In general, the RFID technology enables long-term, 24/7, real-time identification, tracking and general activity measurement of a large number of various animals within a given experimental area (Dudek et al., 2015; Frahm et al., 2018). Its major advantage is the correct long-term identification preservation and traceability of multiple animals in relative complex social housing conditions (Lewejohann et al., 2009; Howerton et al., 2012; Catarinucci et al., 2014; Puścian et al., 2016; Castelhano-Carlos et al., 2017). It can be fully automated to monitor animal’s locomotion activity without human interference, it requires only condensed data storage, and it can easily be integrated with automated monitoring home-cage systems (Catarinucci et al., 2014; Frahm et al., 2018). Since RFID is a detection and tracking technology, its major limitation in the field of animal behavior is the inability to provide detailed information on behavioral traits (grooming, rearing, climbing, etc.). This restricts its application to the sole purpose of animal identification and tracking functionality, which still offers the opportunity to analyze some aspects of social behavior (Puścian et al., 2016; Torquet et al., 2018). Combining RFID with video tracking technology largely extends the possibilities to study social behavior in more detail (see “RFID-Assisted Video Tracking”). Another important technological limitation is the challenge of the RFID antennas to simultaneously detect multiple tags and therefore animals. When multiple animals are located within the reading range of the same RFID antenna, simultaneous signal transmission can interfere with each other, often leading to missed readings and thus data loss. By nature, RFID technology comprises a trade-off between sensitivity (reading range) and accuracy (spatial resolution) since both depend on the dimension of each of the RFID antenna, which is usually the size of the animal in question. The smaller the RFID antenna, the more antennas can be integrated in the RFID antenna matrix underneath the home-cage increasing the positioning and tracking accuracy of the systems and reducing the possibility of signal interference of different RFID tags. On the other hand, small RFID antennas have a shorter reading range. This might result in temporary detection loss in case of vertical movements (i.e., rearing and climbing). This sensitivity-accuracy trade-off needs to be considered when choosing an appropriate RFID hardware set-up and a suitable location of the RFID tag within the animal’s body. Furthermore, RFID is minimally invasive and inserting the tag requires anesthetics. It also bears minor risks of affecting animals’ health condition during long-term application (Albrecht, 2014) or of losing its functionality.

In summary, RFID technology is a great way to identify, track and therefore measure locomotor activity of individual animals in a social context. Its lack of measuring behavioral traits requires the combination with another technology, for example with video tracking.

While there are many companies which provide RFID components, only a few offer an all-in-one and stand-alone apparatus for rodent home-cage application enabling individual voluntary locomotor activity measurements in group-housed settings (Table 1).

**TABLE 1:** Commercially available all-in-one RFID systems for home-cage application.

Company	Product name	Strengths	Limitation	Website	References
PhenoSys	MultimouseMonitor	Multiple animals, small footprint^1^	Limited spatial accuracy, 2 standard sizes available	https://www.phenosys.com/products/multi-mouse-monitor/	Frahm et al., 2018; Alonso et al., 2020
TSE Systems	IntelliCage	Multiple animals (16 mice or 8 rats), small footprint^1^, HCMS containing 4 operant conditioning corners, wide range of cognitive and behavioral tests	Fixed size/dimension, only gross trajectories of individuals	https://www.tse-systems.com/service/intellicage/	Voikar et al., 2018; Kiryk et al., 2020
TSE Systems	TraffiCage	Multiple animals, several sizes available, small footprint^1^	Limited spatial accuracy	https://www.tse-systems.com/service/trafficage/	Dudek et al., 2015; Kotańska et al., 2019
Unified Information Devices	Mouse Matrix	Multiple animals, customizable size/dimension, small footprint^1^, body temperature measurement available	Mice only, not validated yet due to recent market launch	https://www.uidevices.com/home-cage-monitoring/	−

*^1^Footprint refers to how much space the system occupies in addition to the home-cage.*

*Small: Marginal impact on overall space; large: Significant increase of footprint relative to home-cage.*

## Measuring Behavioral Traits

### Infrared Beams

**FIGURE 3:**
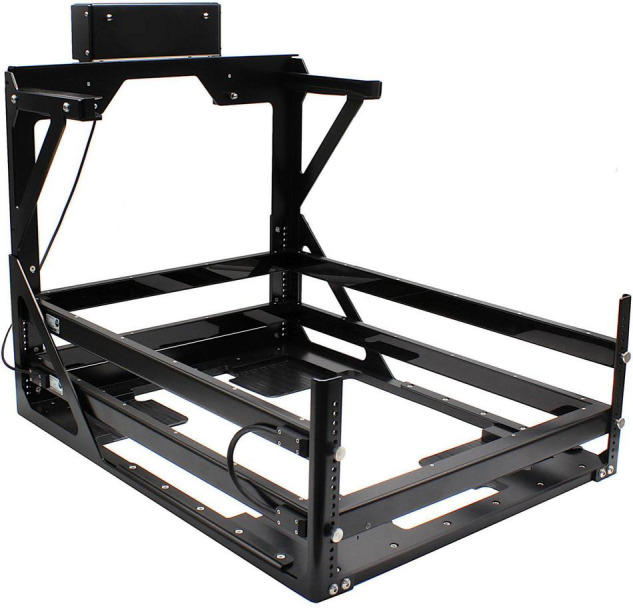
Large infrared beam frame ActiMot3 (TSE Systems) to measure locomotor activity in rodents.

One of the most traditional and simplest principles to continuously monitor voluntary locomotor activity of rodents in the home-cage environment is through infra-red (IR) beam breaks. It uses specially designed frames that surround the home-cage (Figure 3), which emit an array of IR beams invisible to the rodents. Beam interruptions, or breaks, due to the movement of the animal are registered in the horizontal plane (*x*- and *y*-axis) allowing locomotor activity to be reliably detected with a high spatial and temporal resolution. Expanding such systems with an additional frame covering the vertical plane (*z*-axis), furthermore enables the detection and analysis of basic behavioral patterns, such as rearing and climbing. The obtained spatial and temporal information can further be utilized to analyze a variety of other behavioral events, such as feeding and drinking activities (Goulding et al., 2008). Nowadays, associated software packages can fine-tune the raw data to extract a more comprehensive picture of the animal’s behavior, including, but not limited to distance traveled, position distribution, zone entries and trajectory within a given time-period. The use of beam frames is easily applicable, non-invasive, and comes with the freedom to adjust the position of the *z*-frames depending on the desired rodent species and research questions. Since it is independent of lighting conditions, a 24/7 analysis is possible. Numerous home-cages can be simultaneously monitored by the software infrastructure, which generates a clear and small set of raw data without the need of extensive data processing. In general, it can also be easily implemented in automated home-cage monitoring systems (HCMS) to combine behavioral with physiological and metabolic studies (e.g., CLAMS, Columbus Instruments International; Promethion, Sable Systems International; PhenoMaster, TSE Systems). However, a sophisticated behavioral analysis based solely on beam interruptions is difficult due to its limited capability to recognize the rich repertoire of behavioral features that rodents display. Furthermore, to discover the full potential of these beam frames, animals need to be housed individually, otherwise only average group activity can be measured with underestimated activity levels due to blocked beams by the other animals in the same cage. This limits its application for social interaction and behavior studies. Potential occlusion/breaking of the beams by nesting, cage enrichment, or bedding material is another common constraint.

There are several commercially available IR-beam break systems suitable for home-cage application, which mainly differ in accuracy and size (Table 2).

**TABLE 2:** Frequently applied commercially available IR beam frames for home-cage application.

Company	Product name	Strengths	Limitations	Website	References
AfaSci	SmartCage (basic platform module)	Small footprint^1^, extendable with modular add-ons	Animal observation only from top view, resolution unknown	https://www.afasci.com/index.php/instruments/smartcage	Khroyan et al., 2012
Columbus Instruments	Animal Activity Meter: Opto-M4	High flexibility to re-arrange sensors, in-house HCMS component	Large footprint^1^, large spatial resolution (3.12 mm for mice, 6.4 mm for rats), medium temporal resolution (160 Hz)	https://www.colinst.com/products/animal-activity-meter-opto-m	Dorigatti et al., 2021
Kinder Scientific	SmartFrame	Extendable with additional modules, fits several different home-cages	Large footprint^1^, no HCMS component, resolution unknown	http://kinderscientific.com/products/motor-activity-2/cage-rack-2/	Ingram et al., 2013
Omnitech	Custom Home Cage Frame	Interchangeable size for mice/rats, small footprint^1^, max. 60 cages per PC	Max. 2 sensor-axis per cage, resolution unknown, limited scientific validation	https://omnitech-usa.com/product/home-cage/	Reitz et al., 2021
Sable Systems	BXYZ Beam Break Activity Monitor	In-house HCMS component, high spatial (2.5 mm) and temporal (450 Hz) resolution, small footprint^1^	−	https://www.sablesys.com/products/promethion-core-line/promethion-core-cages-and-monitoring/	Woodie et al., 2020
San Diego Instruments	Photobeam Activity System	Small footprint^1^	One size, no HCMS component, low spatial resolution (1.2 cm)	https://sandiegoinstruments.com/product/pas-homecage/	Liu et al., 2021
TSE Systems	ActiMot3	In-house HCMS component, 3 standard sizes (incl. interchangeable for mice/rats), extra high spatial resolution (1.25 mm), small footprint^1^	Medium temporal resolution (100 Hz)	https://www.tse-systems.com/service/actimot3-locomotor-activity/	Neess et al., 2021

*^1^Footprint refers to how much space the system occupies in addition to the home-cage.*

*Small: Marginal impact on overall space; large: Significant increase of footprint relative to home-cage.*

### Force Plates

**FIGURE 4:**
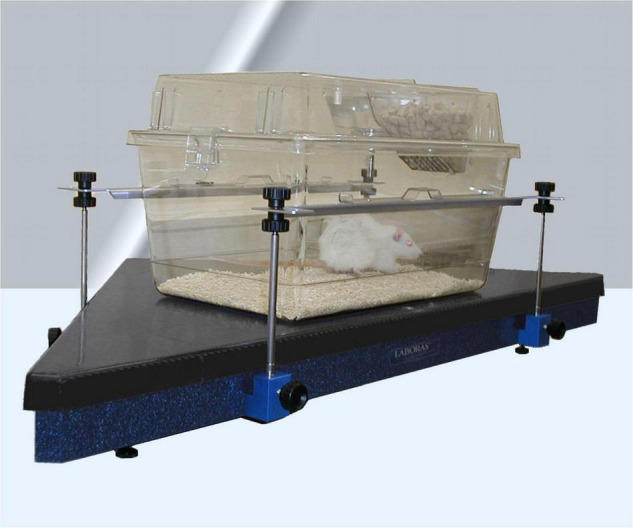
The measurement platform LABORAS turns animal’s movement into electrical signals to analyze an extended set of rodent behavioral patterns (Metris b.v.).

Automated recognition of rodents’ (mice, rats) behavior can also be done by turning mechanical force into electrical signals. Specially designed force plates rest underneath the home-cage and are equipped with sensors that translate the animal’s movement force into electrical signals (Schlingmann et al., 1998; Van de Weerd et al., 2001). Several behavioral attributes can be classified based on their own unique electrical signature characteristics which each of the behavioral traits are generating. The force plates generally enable the quantification of basic behavioral patterns similar to the IR technology, such as resting, rearing, climbing and general locomotion. They also identify the exact position (X,Y) of the animal with high spatiotemporal resolution, providing detailed tracking information, such as trajectories, distance traveled, velocity and position distribution. Probably the most sophisticated force plate system is the Laboratory Animal Behavior Observation Registration and Analysis System (*LABORAS;* Metris b.v., Hoofddorp, Netherlands). It is a specially designed triangular shaped measurement platform (Figure 4). It showed similar results regarding acute locomotor activity compared to IR beam technology (Lynch et al., 2011). Importantly, since the platform recognizes muscle contractions of different body parts (jaws, head, paws, limbs, etc.) more subtle behavioral patterns can also be analyzed (grooming, scratching, seizures, freezing, head shakes, startle response, etc.) which makes this system therefore superior to the IR beam break technology in terms of behavioral profiling. Still, a full sophisticated behavioral analysis is difficult due to the limited discrimination of behavioral patterns in terms of their electrical signatures.

Like the IR beam method, electrical capacitive and RFID technology, force plates can be run fully automated (without human interference/handling), are easily and long-term applicable in mice and rats, non-invasive, independent of lighting conditions, and require relative low handling time. The hardware and software infrastructure enables real-time analysis of multiple platforms at the same time, each generating a small set of raw data. It relies on single housing environments to measure the full set of behavioral patterns thereby limiting its use for social housing conditions. Interestingly, since some social behavioral attributes also generate unique electrical signals, such as for mating or fighting, force plates, in theory, do enable the study of some basic social behavior in pair of rodents. However, force plates are unable to discriminate between conspecifics making it impracticable to attribute these social traits to the individual level.

While the IR beam technology and the force plates generally share many commonalities (similar behavioral parameters) and limitations (most importantly single housing), the LABORAS system identifies a broader range of behavioral patterns, can be used interchangeably for mice and rats, and makes this system thus more versatile in its application. This comes with the limitation of tracking the animal accurately only in a two-dimensional plane (compared to IR frames) as well as of occupying a larger floor space (footprint) due to the wider and more complex construction of the platform.

Currently, there are only a few commercial systems available suitable for home-cage application or already incorporated into a HCMS. These systems mainly differ in the spectrum of behavioral pattern recognition (Table 3).

**TABLE 3:** Available force plates for home-cage applications.

Company	Product name	Strengths	Limitations	Website	References
AfaSci	SmartCage (Vibration sensor module)	Small footprint^1^, extendable with modular add-ons	No stand-alone module, few behavioral parameters (increasable when combined with IR module)	https://www.afasci.com/index.php/instruments/smartcage	Khroyan et al., 2012; Xie et al., 2012
Bioseb	Activmeter	Small footprint^1^, combination with HCMS, rearing/climbing detection optional (mice), numerous behavioral parameters	Few behavioral parameters	https://www.bioseb.com/en/activity-motor-control-coordination/898-activmeter.html	Morgan et al., 2018
Metris	LABORAS	Rich behavioral pattern recognition	Large footprint^1^	https://www.metris.nl/laboras/laboras.htm	Quinn et al., 2003; Castagne et al., 2012
Sable Systems	ADX- Activity Detector	Small footprint^1^, combination with in-house HCMS	Limited behavioral parameters (focus on total activity)	https://www.sablesys.com/products/classic-line/adx-classic-activity-detector/	Fiedler and Careau, 2021
	Home Cage Activity Counter	Adaptable and modifiable do-it-yourself (DIY) system	Limited behavioral parameters (focus on total activity), large footprint^1^	–	Ganea et al., 2007

*^1^Footprint refers to how much space the system occupies in addition to the home-cage.*

*Small: Marginal impact on overall space; large: Significant increase of footprint relative to home-cage.*

Others have integrated piezoelectric sensors into force plates which are able to detect micromovements (Flores et al., 2007). These sensors are generally used to distinguish between sleep and wake phases, serve as an alternative to invasive techniques such as electroencephalograms and electromyograms, and are frequently applied in sleep research (Signal Solutions LLC, Lexington, United States). Piezoelectric sensor technology has introduced new opportunities in behavioral phenotyping and thus gained popularity in the field of animal research. Internal movements such as individual heart beats or breathing cycles are hardly detectable by other phenotyping techniques (including IR-beams or video recording) and the use of piezoelectric sensor plates can thus contribute to establish a more sophisticated rodent ethogram (Carreño-Muñoz et al., 2022).

### Video Tracking

Advances in computational and imaging performance and efficiency have led to new image-based video tracking systems in the field of animal ecology [reviewed by Dell et al. (2014)], which replaces the human observer by a computer to monitor and assess animals’ behavior. Conventionally, these systems consist of hardware and software equipment which undertake a three-way process (Dell et al., 2014). First, the hardware component (one or multiple cameras) digitally records the animals in a given environment and produces a consecutive set of image sequences. Second, the software uses computer vision algorithms to highlight the individual animal from the static background (usually by background subtraction) on each image and propagates its position and thus trajectory across the whole set of images. In group-housed settings, the software must additionally distinguish and separate each individuum from the conspecific (usually creating a pixel blob for each individual) (Giancardo et al., 2013). Individual differences in natural appearance (color, fur pattern, size, contour) serve the software to easier discriminate between individual animals and maintain their identity throughout the video (Hong et al., 2015). Third, the software classifies and quantifies behavioral events based on pre-defined mathematical assumptions established by human expertise (Giancardo et al., 2013). In general, video tracking systems ensure long-term, non-invasive, and real-time tracking of single and multiple animals (Jhuang et al., 2010; Giancardo et al., 2013). By combining optical with IR video, 24/7 tracking is maintained. Video tracking systems enable the analysis of a wide set of behavioral traits with high spatiotemporal resolution and perform well in individual housed rodents (Jhuang et al., 2010). However, two conditions affect the performance and results of such video tracking systems: The complexity of the cage environment and the number of individuals therein, since both result in animals’ (temporal) occlusion from video camera capture. Such occlusion events or animal crossings challenge the software algorithm to preserve the correct animal identity once the individual animal is in sight again or has been separated from its conspecific (Yamanaka and Takeuchi, 2018). This commonly leads to miss identification and/or loss of track, which often propagates throughout the remaining sequence if no appropriate measures are undertaken, i.e., automatic or manual correction (de Chaumont et al., 2012; Giancardo et al., 2013; Yamanaka and Takeuchi, 2018). Marking the animal’s fur with (fluorescent) hair dye or bleach addresses this specific problem (Ohayon et al., 2013; Shemesh et al., 2013), but introduces other drawbacks. Applying artificial markers is time-consuming (needs re-application after some time), requires an invasive procedure (bleaching is done in unconscious animals), and might affect animals’ (social) behavior (Lacey et al., 2007; Dennis et al., 2008). Therefore, different marker-less approaches have been developed aiming to robustly identify and track multiple individuals, such as 3D imaging *via* multiple camera views (Matsumoto et al., 2013), using differences in animals’ body shape/contour (Giancardo et al., 2013), size, color (Noldus et al., 2001; Ohayon et al., 2013; Hong et al., 2015), heat signature (Giancardo et al., 2013), or individual “fingerprints” (Perez-Escudero et al., 2014). Still, accurate and robust maintenance of individual identities within a group remains a major challenge of automated video tracking systems, especially if inbred mice are used which are (almost) indistinguishable by size, color, fur pattern, and likely body shape/contour. The increasing interest in multiple animal tracking with correct identification preservation associated with group-housed conditions has led to a very large number of different video tracking systems and algorithms being available or under development, for a large variety of species and experimental environments [freely available animal tracking software was recently reviewed by Panadeiro et al. (2021)]. Several video tracking systems have already been successfully applied in socially housed rodents, either in an observation cage or open-field arena, usually lacking the supply of drink, feed, and shelter. Examples are *idTracker* (Perez-Escudero et al., 2014), *idtracker.ai* (Romero-Ferrero et al., 2019), *ToxTrac/ToxId* (Rodriguez et al., 2017, 2018), *MiceProfiler* (de Chaumont et al., 2012), *Multi-Animal Tracker* (Itskovits et al., 2017) and *3DTracker* (Matsumoto et al., 2013, 2017). In principle, these systems are suitable for home-cage or social arena applications but require further scientific validation.

Currently, there are only a few systems available or described which are specifically designed for or already validated in rodents socially housed in the home-cage or in a social arena as defined by this review (Table 4). Each of them comes with the strength of analyzing a wide variety of behavioral traits on the individual level within a social context, but also with several different limitations, including, but not limited to, incorrect or loss of identification through animal crossing or visual obstruction (which requires human intervention), low spatial accuracy, limited scientific validation data, facilitating only pairs or small groups, applying artificial markers, or limited data on (social) behavioral parameters.

**TABLE 4:** Overview of image-based video-tracking systems to track and analyze behavioral events in multiple rodents simultaneously in a single home-cage/arena.

Company/Institution/University	Name	Type^1^	Availability	Limitations	Behavioral data output^2^	Max. animals^3^	Identification of unmarked animals	Website	References
California Institute of Technology	Motr	Algorithm	Open source	Invasive, manual correction required, limited behavioral pattern recognition	Social	6	No (bleaching)	https://motr.janelia.org/	Ohayon et al., 2013
California Institute of Technology	–	All-in-one	DIY	Different fur color required	Social	2	Yes (different fur color required)	–	Hong et al., 2015
CleverSys	GroupHousedScan	Software^4^	Commercial	Not validated yet	Individual + social	–/4	–	http://cleversysinc.com/CleverSysInc/csi_products/grouphousedscan/	–
Hiroshima University	UMATracker	Software	Open Source	Manual correction required after identity swap	Social	4	Yes	https://ymnk13.github.io/UMATracker/	Yamanaka and Takeuchi, 2018
Loligo Systems	LoliTrack 5	Software	Commercial	Not validated in rodents yet	Individual + limited social		Yes	https://loligosystems.com/lolitrack-version-5-video-tracking-and-behavior-analysis-software	–
National Institute of Health	SCORHE	All-in-one	Open source (software)	Individual identities not maintained, mice only	Individual (grouped)	2^5^	Yes	https://spis.cit.nih.gov/node/30	Salem et al., 2015
Noldus	EthoVision/PhenoTyper	All-in-one	Commercial	Marking required	Individual + social	5/16	No (color marking)	https://www.noldus.com/ethovision-xt/https://www.noldus.com/phenotyper	Noldus et al., 2001; de Visser et al., 2006
University of California, San Diego	Smart Vivarium	Algorithm	DIY	Poor identity maintenance after occlusion	Individual	3^5^	Yes	http://smartvivarium.calit2.net/	Branson and Belongie, 2005
Weizmann Institute of Science	–	All-in-one	DIY	Color marking	Social	4/ > 10	No (color marking)	–	Shemesh et al., 2013

*^1^All-in-one: one apparatus consisting of complete hardware and software infrastructure without the need of additional equipment to be purchased.*

*–Software: software package with a graphical user interface.*

*–Algorithm: source codes which can be implemented in software solutions or require external programming and analyzing platforms, such as MATLAB or Icy.*

*^2^Individual: measurement of individual behavioral traits.*

*–Social: measurement of social interaction.*

*^3^Maximum number of rodents which has been scientifically validated in the mentioned reference(s)/officially communicated by the developer of the system.*

*^4^Associated hardware equipment available from same vendor.*

*The full power of the system (tracking + behavioral phenotyping) requires solitary housing.*

Recently, artificial intelligence has become very prominent on different aspects of computer vision technology and enabled such systems to learn from existing data (Rousseau et al., 2000). Nowadays, most of the computer vision systems have incorporated at least some elements of artificial intelligence, starting from animal detection toward the automated analysis of behavioral traits. In general, machine learning approaches are applied in a supervised fashion, meaning that the existing training video sequences were first labeled and then classified into specific behavioral traits by human experts. This required the systems to be programmed by humans in order to set robust rules for identifying specific behavioral attributes.

**FIGURE 5:**
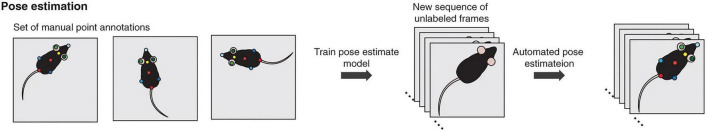
Machine learning approach of pose estimation (von Ziegler et al., 2021).

A new promising approach within the field of machine learning is inspired by human joint localization (Toshev and Szegedy, 2014), which enables the tracking of joints or body parts and thereby measures different postures. This approach is rapidly finding its way into laboratory settings using the tracking of multiple body parts to establish postures. Indeed, posture estimation algorithms have been developed tailoring the “human approach” to laboratory animals. For example, *DeepLabCut* (Mathis et al., 2018; Nath et al., 2019) and *LEAP Estimates Animal Pose* (LEAP) (Pereira et al., 2019) are based on algorithms previously applied in humans (Insafutdinov et al., 2016). In general, these algorithms use a three-step process: First, specific body parts (joints or key points) of interest are manually labeled on selected video images. Second, the pose estimation model is trained to recognize the corresponding body parts. Third, the trained algorithm is applied to the full video sequence for automatic prediction of body part location and thus pose estimation (Figure 5).

In contrast to conventional machine learning technology which often focusses on tracking only the centroid of each animal, these new algorithms provide tracking of multiple body parts. Therefore, the main advantage of these body-part-algorithms is the analysis of a tremendous variety of behavioral patterns, postures and orientation in various animals based on a limited training period. No visual marking of the animal is required, it is non-invasive, freely available, open-source and thus gives the researcher the freedom to adjust the algorithm to the particular needs. Furthermore, analyzing the raw data set once the experiment (or video) is completed, offers *post hoc* analysis of specific scientific questions. The commonly observed speed-accuracy trade-off generally experienced in the field of machine learning, has been solved recently (Graving et al., 2019). A drawback may be that specific postures (based on user-defined body parts) need to be predefined before applying the algorithm, which may induce laboratory or investigator specific variation. Also, training the algorithm requires manual annotation, which is, even on a small set of video images, labor intensive. The need to train the algorithm on individual animals often prevents real-time analysis. Interestingly, recent developments in the field provide real-time approaches based on already trained data sets (Kane et al., 2020). LEAP requires the least amount of training images and—like DeepLabCut—has currently been optimized for group-housed conditions in order to identify individual animals in a social group, resulting in *Social LEAP Estimates Animal Pose* (SLEAP) (Pereira et al., 2020; Lauer et al., 2021). However, analysis of multiple animals is prone to visual occlusion and can be very laborious (requires annotation of every individual per image), especially when analysis of complex postures is desired. Nevertheless, DeepLabCut has been shown to outperform commercially available systems regarding animal tracking and is able to compete with human scoring of relevant behavioral patterns (Sturman et al., 2020).

In summary, the use of such algorithms is representative of a new generation of video tracking systems within the rapidly evolving field of behavioral animal research. Current algorithm development focusses on pose-estimation and body part classification of unmarked animals enabling the analysis of various predefined body postures and behavioral patterns. On the other hand, the open-source, publicly accessible software/algorithm needs to be combined with (commercially available) hardware infrastructure to conduct video tracking. For each experiment, manual labeling of predefined images is still required which can be prone to subjectivity. It can also be a laborious process, especially during social experiments.

### RFID-Assisted Video Tracking

The biggest challenge of applying video tracking technology is maintaining the correct identification and thus position and direction of multiple interacting animals. To solve this problem to the best, the strength of the RFID technology (consistent identification of almost unlimited numbers of animals even in diverse complex living environments) has been combined with the strengths of video tracking (high spatiotemporal analysis of complex (social) behavioral events) resulting in a synergistic hybrid tracking technology (Weissbrod et al., 2013; Dandan and Lin, 2016).

**FIGURE 6:**
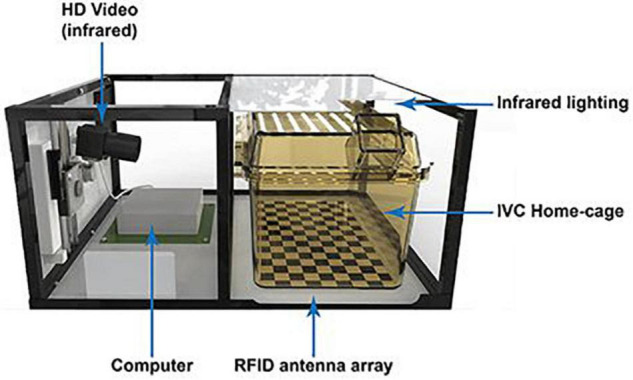
The Home Cage Analyser (Actual Analytics) combines RFID technology with video recording to study behavioral traits in socially interacting rodents (Bains et al., 2016).

The *Home Cage Analyser* (Actual Analytics Ltd., Edinburgh, United Kingdom) (Figure 6; Bains et al., 2016; Redfern et al., 2017) and the *RFID-Assisted SocialScan* (CleverSys Inc., Reston, United States) (Peleh et al., 2019) are commercially available systems that integrate RFID tracking with 2D IR video capturing. By synchronizing the RFID readings with the video tracking, possible identity swaps are automatically corrected by the software without human intervention. The *Live Mouse Tracker* is similar to the aforementioned systems but uses a depth-sensing camera for three-dimensional activity and behavior monitoring of multiple mice in a social arena (de Chaumont et al., 2019). Its main advantage is a very rich repertoire of 35 behavioral patterns that can be recognized—again, without the need of human intervention. The analysis ranges from simple locomotor activity of individual mice toward more sophisticated social behavior between multiple (*n* = 4) conspecifics. Furthermore, it is a comprehensive, do-it-yourself, and end-to-end solution based on open-source frameworks. At present, the Live Mouse Tracker sets a new standard in multiple animal phenotyping, since it offers an open-source end-to-end solution, is easy to apply for an ordinary researcher, and—importantly—enables the analysis of a considerable set of behavioral patterns supported by machine learning, however, currently in mice only.

In conclusion, RFID-assisted video tracking systems combine the strengths of video tracking and RFID technology to create a synergistic effect. These systems overcome major limitations of the previous listed technologies making it possible to continuously track many individual rodents including monitoring and quantifying individual as well as social behavioral traits in a complex environment. Since these systems have become available to the market rather recently, further developments on hardware and especially software solutions (machine learning) will certainly enhance the performance and wider applications of these hybrid tracking systems (Table 5).

**TABLE 5:** Available RFID-assisted video tracking technology.

Company/Institute	Product name	Environment	Characteristics	Website	References
Actual Analytics	Home Cage Analyser	Home Cage	Side-view camera (prone to occlusion), all-in-one apparatus, mice and rats, individual and social parameters, relatively small footprint^1^	https://www.actualanalytics.com/products	Bains et al., 2016; Redfern et al., 2017; Hobson et al., 2020
Clever Sys Inc.	RFID-Assisted SocialScan	Social arena	Top-view camera, all-in-one apparatus, mice and rats, rodents need to be distinguishable by color/size, Focus on social behavior parameters, small footprint^1^	http://cleversysinc.com/CleverSysInc/rfid-assisted-socialscan/	Peleh et al., 2019; Peleh et al., 2020
Institute Pasteur	Live Mouse Tracker	Social arena	Top-view camera, DIY, mice only, individual and rich social behavioral parameters, small footprint^1^, end user can add new behavioral parameters of interest	https://livemousetracker.org/	Ey et al., 2018; de Chaumont et al., 2019

*^1^Footprint refers to how much space the system occupies in addition to the home-cage/social arena.*

*Small: Marginal impact on overall space; large: Significant increase of footprint relative to cage environment.*

## Summary

**FIGURE 7:**
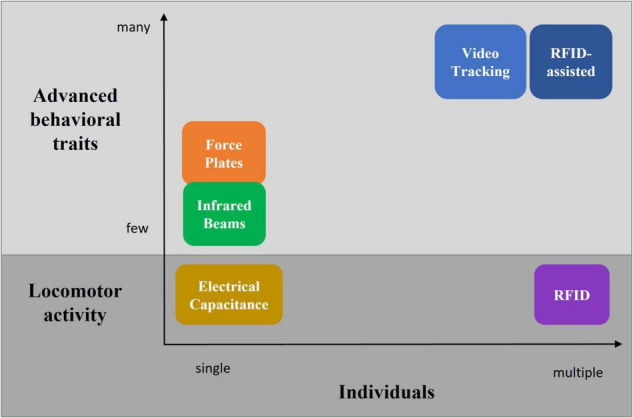
Overview of different technologies to measure locomotor activity and advanced behavioral traits in individually and group-housed rodents.

There are a fair number of different systems available for behavioral phenotyping of rodents living in home-cages or social arenas (Figure 7 and Table 6). These range from targeting voluntary locomotor activity measurements toward more advanced methods which expand the analysis of the behavioral repertoire beyond basic locomotor activity metrics. These methods often comprise a trade-off between group housing and extended behavioral pattern recognition. A well-established and prominent method of behavior analysis is the use of video tracking systems, especially in combination with recent advances in machine learning technology. Unfortunately, until now only a minority of such systems have been validated in a social context and in meaningful and heterogeneous environments, such as the home-cage, consisting of appropriate refinement material. The latter challenges the performance of dedicated tracking and behavioral phenotyping systems. Latest developments in multiple pose estimation hold great promise in further enhancing the performance of such video tracking systems. Importantly, a common technical limitation of video-tracking systems is the correct identification preservation, which is compromised by animal crossing or camera occlusion. Prevention requires human intervention and thus prohibits large-scale, high-throughput studies. Combining the strengths of video tracking and RFID technology opens the door to a much more complex analysis of locomotor activity and behavioral traits of socially interacting animals. At the same time, it addresses the identification preservation challenge. Therefore, such RFID-assisted video tracking solutions seem to be the most comprehensive systems currently available and hold great promise for further development.

**TABLE 6:** Overview of strengths and limitations of current technology used to measure rodents’ locomotor activity and more advanced behavioral aspects in the home-cage.

Technology	Housing	Advanced behavioral traits^1^	Strengths	Limitation
Electrical capacitance	Individual^2^	No	High spatial accuracy, small (data) footprint	Single housing, no behavioral parameters (only locomotor activity), no stand-alone method, mice only
RFID	Individual/Group	No	Social housing, reliable animal identification and tracking even with high animal density, small (data) footprint	Low spatial resolution, no behavioral parameters (only locomotor activity), possible data loss due to animal interferences
Infrared beam	Individual^2^	Few	High spatiotemporal accuracy, small (data) footprint	Single housing, few behavioral parameters
Force plate	Individual^2^	Few	Small (data) footprint	Single housing, few behavioral parameters
Video tracking	Individual/Group	Many	Social housing, rich (social) behavioral pattern recognition	Frequent identity swaps require corrections, high processing power, large data footprint
RFID-assisted video tracking	Individual/Group	Many	Social housing, rich (social) behavioral pattern recognition, large animal density	High processing power, large (data) footprint

*^1^Behavioral traits which go beyond basic locomotor activity metrics.*

*^2^Group-housing possible, but would limit the full scope of the technology.*

## Future Perspective

Recent developments in computer vision have resulted in several freely available open-source software and algorithm solutions that can be shared among the scientific community for user-defined application and further development. The use and development of open-source software is encouraged by the European Commission to foster innovation by sharing knowledge and expertise (European Commission, 2020). It also allows insight into how data are processed by the software and consequences of changes can be better understood. The ambition to improve animal tracking is further enhanced by current trends in the field of machine learning, which is rapidly gaining ground in animal research (von Ziegler et al., 2021). This development will continue to increase the supply of new software solutions freely available for the research community (Nilsson et al., 2020; Hsu and Yttri, 2021), providing alternatives to costly and commercially available tracking software packages. Importantly, machine learning algorithms have already proven to outperform commercially available systems regarding animal tracking, highlighting their promising capability for future applications (Sturman et al., 2020).

Most of these machine learning algorithms still require individually housed animals (Wiltschko et al., 2015; Geuther et al., 2019; Pennington et al., 2019). DeepLabCut and SLEAP act as forerunner to more complex situations, inspiring others to follow (Mathis et al., 2018; Pereira et al., 2020). Despite their reliance on training the algorithm by human annotations, machine learning algorithms have drastically reduced the need and time of human labeling compared to manual scoring (either real-time or post video tracking) (Jhuang et al., 2010; van Dam et al., 2013). Once trained by an individual or a group of experts, the algorithm replicates the human input on any new data sets. Thereby the inter-observer variability is diminished within and across laboratories, which contributes to objectivity and consistency and thus reproducibility and replicability of scientific data (Levitis et al., 2009). At present, human annotations set the benchmark for such automated systems to be able to recognize, classify and thus quantify specific behavioral events. To overcome the *human factor* and to push machine learning into a new direction, there are recent ambitious efforts to optimize unsupervised machine learning methods (Todd et al., 2017). Such algorithms ensure behavioral classification in an unsupervised manner which makes the need of annotation of several example images by a human expert redundant. One such promising new algorithm is *AlphaTracker.* It achieves behavioral classification of individual as well as social behavioral motifs of identical and unmarked mice with high accuracy aiming to overcome the commonly identification preservation challenge in socially housed animals (Chen et al., 2020). The wide application of those type of algorithms might revolutionize our current understanding of (rodent) animal behavior, since very subtle and unexpected behavioral events (“syllables”) can be analyzed and studied in more detail, which are unrecognizable for the human eye (Wiltschko et al., 2015; Markowitz et al., 2018). Such potential new behavioral traits need to be classified in a way which reaches consensus among the behavioral scientific community supporting the interpretation as well as the reproducibility and replicability of research data. Interestingly, unsupervised algorithms are currently under development, tailored to combine behavioral analysis and electrophysiological recordings. The algorithm’s properties are fine-tuned to meet the specific requirements (i.e., high temporal resolution) for analyzing electrophysiological characteristics during behavioral studies (Hsu and Yttri, 2021).

One of the drawbacks associated with the general use of open-source machine learning technology is the necessity for the user to have at least some basic, if not substantial, computational expertise. This can serve as a high entry barrier for research laboratories to implement and further develop such methods, especially for non-behavioral research groups aiming for interdisciplinary breakthroughs. Associated video equipment is often less flexible to be incorporated into HCMS. These issues have already been addressed by some developers and need to be taken into account to make an innovative technology user-friendly and thus widely applicable in practice (Mathis et al., 2018; Singh et al., 2019; Nilsson et al., 2020). Commercially available all-in-one solutions come with a higher financial burden but are generally more user-friendly and thus lower such entry barrier significantly. They also include customer support to assist laboratories to conduct their research in a technologically sound way. In the end, the regular user will decide whether to rely on more financially demanding, but sophisticated and technically mature all-in-one solutions or to step toward more flexible, but computational resource-depending open-software and -hardware applications.

## Author Contributions

CK wrote the manuscript. TB, JH, DV, JK, and ES revised and edited the manuscript. All authors approved the submitted version.

## Conflict of Interest

CK, TB, and DV were employed by TSE Systems GmbH. The remaining authors declare that the research was conducted in the absence of any commercial or financial relationships that could be construed as a potential conflict of interest.

## Publisher’s Note

All claims expressed in this article are solely those of the authors and do not necessarily represent those of their affiliated organizations, or those of the publisher, the editors and the reviewers. Any product that may be evaluated in this article, or claim that may be made by its manufacturer, is not guaranteed or endorsed by the publisher.
