# Corrigendum: Measuring Locomotor Activity and Behavioral Aspects of Rodents Living in the Home-Cage

**DOI:** 10.3389/fnbeh.2022.943307

**Published:** 2022-06-20

**Authors:** Christian J. M. I. Klein, Thomas Budiman, Judith R. Homberg, Dilip Verma, Jaap Keijer, Evert M. van Schothorst

**Affiliations:** ^1^Human and Animal Physiology, Wageningen University and Research, Wageningen, Netherlands; ^2^TSE Systems GmbH, Berlin, Germany; ^3^Department of Cognitive Neuroscience, Donders Institute for Brain, Cognition and Behavior, Radboud University Medical Center, Nijmegen, Netherlands

**Keywords:** locomotor activity, behavior, home-cage, rodents, 3Rs, phenotyping, animal tracking

In the original article, there was an error in *Measuring Voluntary Locomotor Activity, Electrical Capacitance, Paragraph 1*. The spatial resolution was given in cm instead of mm and the word “currently” was missing in the sentence “This makes this system unable to study social interaction and behavior.” The revised paragraph appears below:

Measuring an animal's activity can be done by electrical capacitance technology. This technology comprises several electrodes embedded in an electronic sensing board (**Figure 1**), which is installed underneath the home-cage. The animal's presence changes the electromagnetic field emitted by these electrodes. Thereby, the exact position (with spatial resolution of 1 mm) and trajectory can be identified based on capacity variation [with temporal resolution of 4 hertz (Hz)]. The sensing board sends its raw data to an associated software and computer infrastructure, which enables the researcher to additionally analyze distance traveled, average speed, position distribution, and activity density of the animal. The activity metrics show comparable results when benchmarked against video-recording technology (Iannello, 2019). This board was developed as part of the Digital Ventilated Cage (DVC) monitoring system (Tecniplast, Buguggiate, Italy), allowing fully automated, 24/7, non-invasive, real-time activity monitoring and traceability of individually housed mice. It requires only modest computational power resulting in a small data footprint per unit. It is highly scalable, allowing arbitrary numbers of home-cages to be monitored simultaneously. DVC-derived datasets can be used subsequently for a deeper analysis of several activity metrics in individual-housed mice (Shenk et al., 2020). However, this system does not support the analysis of ethologically relevant behavioral patterns (grooming, rearing, climbing etc.) which makes it less suitable for phenotyping and behavioral studies. It is currently also designed for the use of mice only. Whereas multiple animals can be housed in one home-cage to monitor group activity (Pernold et al., 2019), the full potential of the technology relies on individually housed conditions. This makes this system currently unable to study social interaction and behavior. Since it was originally developed as a component of the DVC system, it cannot be integrated in automated monitoring systems of other vendors. In conclusion, the sensor plate is a useful module within the DVC system aiming to improve animals' health monitoring and facility management. It allows monitoring of overall activity, but the limited behavioral pattern recognition makes this system less suitable for more sophisticated phenotyping and behavioral studies, especially in group-housed settings.

The authors apologize for this error and state that this does not change the scientific conclusions of the article in any way. The original article has been updated.

## Publisher's Note

All claims expressed in this article are solely those of the authors and do not necessarily represent those of their affiliated organizations, or those of the publisher, the editors and the reviewers. Any product that may be evaluated in this article, or claim that may be made by its manufacturer, is not guaranteed or endorsed by the publisher.
